# 4,5-Dichloro-2-methyl­pyridazin-3(2*H*)-one

**DOI:** 10.1107/S1600536809046947

**Published:** 2009-11-11

**Authors:** Jia Hao Goh, Hoong-Kun Fun, P. V. R. Rukmini, B. Kalluraya

**Affiliations:** a X-ray Crystallography Unit, School of Physics, Universiti Sains Malaysia, 11800 USM, Penang, Malaysia; b Department of Studies in Chemistry, Mangalore University, Mangalagangotri, Mangalore 574 199, India

## Abstract

The asymmetric unit of the title compound, C_5_H_4_Cl_2_N_2_O, contains one half-mol­ecule: all the non-H atoms lie on a crystallographic mirror plane. In the crystal structure, mol­ecules are linked into chains along the *c* axis by weak inter­molecular C—H⋯O hydrogen bonds.

## Related literature

For general background to and applications of pyridazine derivatives, see: Banerjee *et al.* (2009 ▶); Samuel & Bose (1987 ▶); Siddiqui & Wani (2004 ▶). For standard bond lengths, see: Allen *et al.* (1987 ▶). For the stability of the temperature controller used for the data collection, see: Cosier & Glazer (1986 ▶).
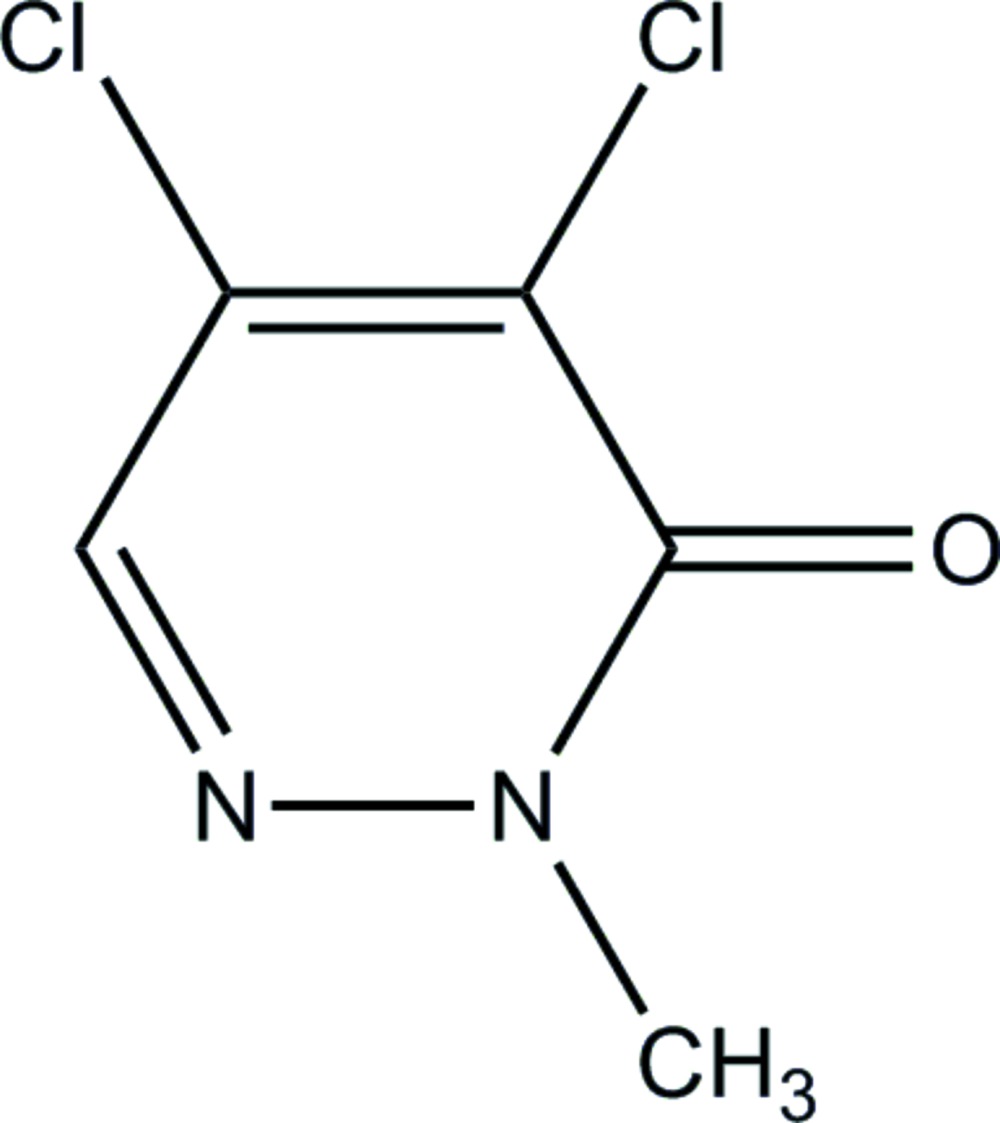


## Experimental

### 

#### Crystal data

C_5_H_4_Cl_2_N_2_O
*M* *_r_* = 179.00
Orthorhombic, 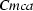

*a* = 6.5157 (1) Å
*b* = 15.9127 (4) Å
*c* = 13.5175 (3) Å
*V* = 1401.53 (5) Å^3^
*Z* = 8
Mo *K*α radiation
μ = 0.85 mm^−1^
*T* = 100 K
0.42 × 0.29 × 0.22 mm

#### Data collection

Bruker SMART APEXII CCD area-detector diffractometer
Absorption correction: multi-scan (**SADABS**; Bruker, 2005 ▶) *T*_min_ = 0.717, *T*_max_ = 0.837
13402 measured reflections
1659 independent reflections
1427 reflections with *I* > 2σ(*I*)
*R*_int_ = 0.029

#### Refinement

*R*[*F* ^2^ > 2σ(*F* ^2^)] = 0.030
*wR*(*F* ^2^) = 0.085
*S* = 1.09
1659 reflections
64 parameters
H atoms treated by a mixture of independent and constrained refinement
Δρ_max_ = 0.55 e Å^−3^
Δρ_min_ = −0.40 e Å^−3^

### 

Data collection: *APEX2* (Bruker, 2005 ▶); cell refinement: *SAINT* (Bruker, 2005 ▶); data reduction: *SAINT*; program(s) used to solve structure: *SHELXTL* (Sheldrick, 2008 ▶); program(s) used to refine structure: *SHELXTL*; molecular graphics: *SHELXTL*; software used to prepare material for publication: *SHELXTL* and *PLATON* (Spek, 2009 ▶).

## Supplementary Material

Crystal structure: contains datablocks global, I. DOI: 10.1107/S1600536809046947/lh2946sup1.cif

Structure factors: contains datablocks I. DOI: 10.1107/S1600536809046947/lh2946Isup2.hkl

Additional supplementary materials:  crystallographic information; 3D view; checkCIF report

## Figures and Tables

**Table 1:** Hydrogen-bond geometry (Å, °)

*D*—H⋯*A*	*D*—H	H⋯*A*	*D*⋯*A*	*D*—H⋯*A*
C4—H4*A*⋯O1^i^	0.980 (19)	2.328 (19)	3.2988 (18)	170.6 (16)

Symmetry code: (i) 
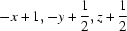
.

**Fig. 1.**
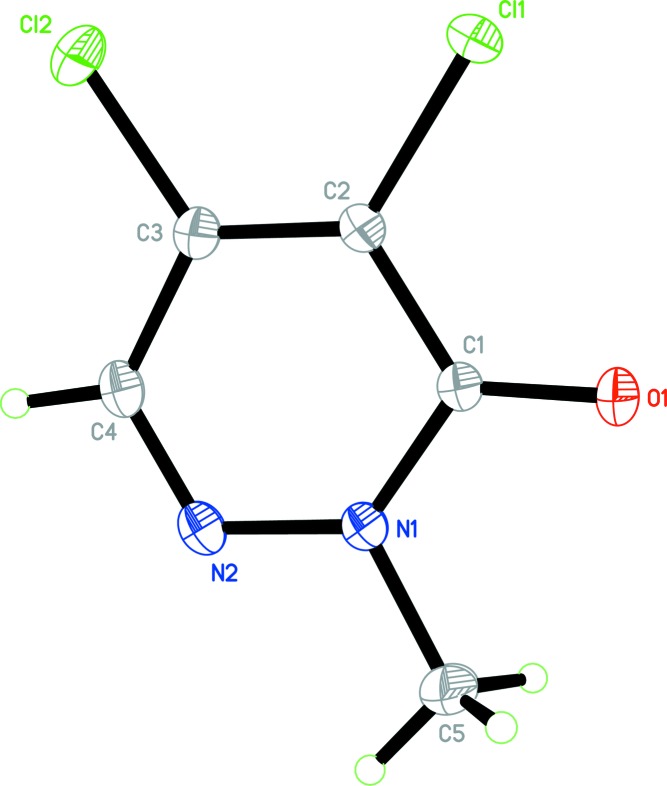
The molecular structure of the title compound, showing 50% probability displacement ellipsoids for non-H atoms and the atom-numbering scheme [symmetry code: 1-x, y, z for one methyl hydrogen atom not lying on the mirror plane].

**Fig. 2.**
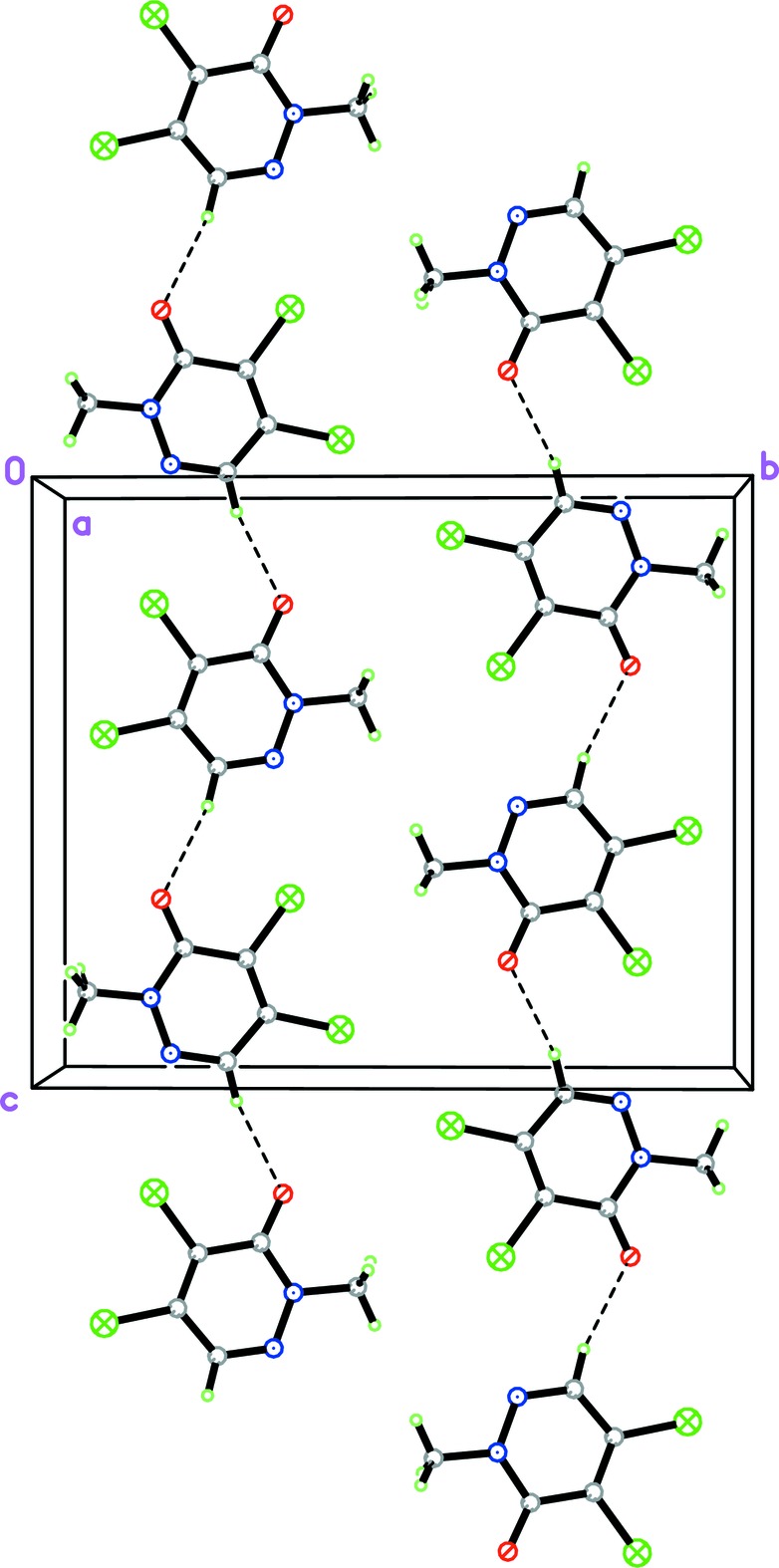
Part of the crystal structure of the title compound viewed along the a axis, showing one-dimensional chains along the c axis. Hydrogen bonds are shown as dashed lines.

## supplementary crystallographic information

### Comment

Pyridazin-3(2*H*)-one derivatives represent one of the most active class of compounds possessing a wide spectrum of biological activities such as cardiovascular properties, anti-inflammatory, anti-diabetic, analgesic, anti-AIDS, anti-cancer, anti-microbial and anti-convulsant activities (Banerjee *et al.*, 2009; Siddiqui & Wani, 2004). Effects of substituted pyridazinones on photosynthetic electron transport have been studied by various workers and are known to inhibit photosystem II (PS II) electron transport (Samuel & Bose, 1987). Herein we report the crystal structure of the title compound.

The asymmetric unit of the title compound contains one half-molecule and all atoms, with the exception of one methyl hydrogen atom [symmetry related H atom generated by 1-x, y, z], lie on a crystallographic mirror plane (Fig. 1). The bond lengths (Allen *et al.*, 1987) and angles are within normal ranges. In the crystal structure (Fig. 2), neighbouring molecules are linked into one-dimensional chains along the *c* axis by intermolecular C4—H4A···O1^i^ hydrogen bonds (Table 1).

### Experimental

4,5-dichloropyridazin-3(2*H*)-one (0.01 mol) and methanol (9.7 ml) was placed into a R.B. flask. The contents were stirred for 15 minutes. Sodium hydroxide (0.5 g) in de-mineralized water (10.0 ml) was added with constant stirring. As a clear solution is observed, the R.B. flask was cooled to 278 K. When the temperature fell below 278 K, dimethyl sulphate (0.01 mol) was added dropwise. Stirring was continued, maintaining the temperature between 288–293 K over 1 h. Excess methanol was distilled off under reduced pressure. The solid obtained was collected by filtration, washed with water and dried. The crude product obtained was purified by recrystallization from ethanol. Single crystals suitable for X-ray analysis were obtained recrystallization from a 1:2 mixture of DMF and ethanol by slow evaporation.

### Refinement

The hydrogen atom H4A was located from difference Fourier map and allowed to refine freely. The hydrogen atoms bound to atom C5 were located geometrically and refined using a riding model with C—H = 0.96 Å and *U*_iso_(H) = 1.5 *U*_eq_(C).

### Crystal data

**Table:**

C_5_H_4_Cl_2_N_2_O	*F*(000) = 720
*M**_r_* = 179.00	*D*_x_ = 1.697 Mg m^−^^3^
Orthorhombic, *C**m**c**a*	Mo *K*α radiation, λ = 0.71073 Å
Hall symbol: -C 2bc 2	Cell parameters from 6513 reflections
*a* = 6.5157 (1) Å	θ = 2.6–34.7°
*b* = 15.9127 (4) Å	µ = 0.85 mm^−^^1^
*c* = 13.5175 (3) Å	*T* = 100 K
*V* = 1401.53 (5) Å^3^	Block, colourless
*Z* = 8	0.42 × 0.29 × 0.22 mm

### Data collection

**Table:**

Bruker SMART APEXII CCD area-detector diffractometer	1659 independent reflections
Radiation source: fine-focus sealed tube	1427 reflections with *I* > 2σ(*I*)
graphite	*R*_int_ = 0.029
φ and ω scans	θ_max_ = 35.0°, θ_min_ = 2.6°
Absorption correction: multi-scan (*SADABS*; Bruker, 2005)	*h* = −10→10
*T*_min_ = 0.717, *T*_max_ = 0.837	*k* = −25→24
13402 measured reflections	*l* = −20→21

### Refinement

**Table:**

Refinement on *F*^2^	Primary atom site location: structure-invariant direct methods
Least-squares matrix: full	Secondary atom site location: difference Fourier map
*R*[*F*^2^ > 2σ(*F*^2^)] = 0.030	Hydrogen site location: inferred from neighbouring sites
*wR*(*F*^2^) = 0.085	H atoms treated by a mixture of independent and constrained refinement
*S* = 1.09	*w* = 1/[σ^2^(*F*_o_^2^) + (0.0513*P*)^2^ + 0.3383*P*] where *P* = (*F*_o_^2^ + 2*F*_c_^2^)/3
1659 reflections	(Δ/σ)_max_ < 0.001
64 parameters	Δρ_max_ = 0.55 e Å^−^^3^
0 restraints	Δρ_min_ = −0.40 e Å^−^^3^

### Special details

**Table:**

Experimental. The crystal was placed in the cold stream of an Oxford Cyrosystems Cobra open-flow nitrogen cryostat (Cosier & Glazer, 1986) operating at 100.0 (1)K.
Geometry. All esds (except the esd in the dihedral angle between two l.s. planes) are estimated using the full covariance matrix. The cell esds are taken into account individually in the estimation of esds in distances, angles and torsion angles; correlations between esds in cell parameters are only used when they are defined by crystal symmetry. An approximate (isotropic) treatment of cell esds is used for estimating esds involving l.s. planes.
Refinement. Refinement of F^2^ against ALL reflections. The weighted R-factor wR and goodness of fit S are based on F^2^, conventional R-factors R are based on F, with F set to zero for negative F^2^. The threshold expression of F^2^ > 2sigma(F^2^) is used only for calculating R-factors(gt) etc. and is not relevant to the choice of reflections for refinement. R-factors based on F^2^ are statistically about twice as large as those based on F, and R- factors based on ALL data will be even larger.

### Fractional atomic coordinates and isotropic or equivalent isotropic displacement parameters (Å^2^)

**Table:**

	*x*	*y*	*z*	*U*_iso_*/*U*_eq_	
Cl1	0.5000	0.152043 (19)	0.19640 (2)	0.01834 (9)	
Cl2	0.5000	0.07981 (2)	0.41860 (3)	0.02540 (10)	
O1	0.5000	0.33799 (6)	0.19775 (7)	0.0221 (2)	
N1	0.5000	0.35316 (6)	0.36546 (8)	0.0182 (2)	
N2	0.5000	0.32435 (8)	0.45961 (9)	0.0194 (2)	
C1	0.5000	0.30554 (8)	0.28034 (9)	0.0159 (2)	
C2	0.5000	0.21504 (8)	0.29869 (10)	0.0152 (2)	
C3	0.5000	0.18537 (8)	0.39241 (10)	0.0173 (2)	
C4	0.5000	0.24313 (9)	0.47317 (11)	0.0190 (2)	
C5	0.5000	0.44471 (9)	0.35542 (13)	0.0296 (3)	
H5A	0.5000	0.4700	0.4199	0.044*	
H5B	0.6203	0.4621	0.3199	0.044*	
H4A	0.5000	0.2257 (12)	0.5427 (14)	0.016 (4)*	

### Atomic displacement parameters (Å^2^)

**Table:**

	*U*^11^	*U*^22^	*U*^33^	*U*^12^	*U*^13^	*U*^23^
Cl1	0.02167 (15)	0.01747 (14)	0.01587 (16)	0.000	0.000	−0.00382 (10)
Cl2	0.03383 (19)	0.01782 (15)	0.02456 (19)	0.000	0.000	0.00717 (11)
O1	0.0344 (6)	0.0193 (4)	0.0124 (4)	0.000	0.000	0.0030 (3)
N1	0.0257 (5)	0.0156 (4)	0.0133 (5)	0.000	0.000	−0.0007 (4)
N2	0.0235 (5)	0.0218 (5)	0.0129 (5)	0.000	0.000	−0.0014 (4)
C1	0.0192 (5)	0.0152 (5)	0.0132 (5)	0.000	0.000	0.0005 (4)
C2	0.0165 (5)	0.0156 (5)	0.0134 (5)	0.000	0.000	−0.0007 (4)
C3	0.0193 (5)	0.0173 (5)	0.0155 (5)	0.000	0.000	0.0019 (4)
C4	0.0214 (5)	0.0224 (6)	0.0132 (6)	0.000	0.000	0.0012 (4)
C5	0.0503 (10)	0.0160 (5)	0.0224 (7)	0.000	0.000	−0.0012 (5)

### Geometric parameters (Å, °)

**Table:**

Cl1—C2	1.7078 (13)	C1—C2	1.4613 (18)
Cl2—C3	1.7166 (13)	C2—C3	1.3520 (19)
O1—C1	1.2301 (15)	C3—C4	1.427 (2)
N1—N2	1.3528 (16)	C4—H4A	0.980 (18)
N1—C1	1.3778 (16)	C5—H5A	0.9599
N1—C5	1.4631 (17)	C5—H5B	0.9600
N2—C4	1.3053 (19)		
			
N2—N1—C1	126.82 (11)	C2—C3—C4	119.46 (12)
N2—N1—C5	115.13 (11)	C2—C3—Cl2	122.34 (11)
C1—N1—C5	118.04 (11)	C4—C3—Cl2	118.20 (11)
C4—N2—N1	117.88 (11)	N2—C4—C3	122.03 (13)
O1—C1—N1	121.81 (11)	N2—C4—H4A	114.5 (11)
O1—C1—C2	124.60 (11)	C3—C4—H4A	123.5 (11)
N1—C1—C2	113.59 (11)	N1—C5—H5A	109.5
C3—C2—C1	120.22 (12)	N1—C5—H5B	109.5
C3—C2—Cl1	123.62 (10)	H5A—C5—H5B	109.5
C1—C2—Cl1	116.16 (9)		
			
C1—N1—N2—C4	0.0	N1—C1—C2—Cl1	180.0
C5—N1—N2—C4	180.0	C1—C2—C3—C4	0.0
N2—N1—C1—O1	180.0	Cl1—C2—C3—C4	180.0
C5—N1—C1—O1	0.0	C1—C2—C3—Cl2	180.0
N2—N1—C1—C2	0.0	Cl1—C2—C3—Cl2	0.0
C5—N1—C1—C2	180.0	N1—N2—C4—C3	0.0
O1—C1—C2—C3	180.0	C2—C3—C4—N2	0.0
N1—C1—C2—C3	0.0	Cl2—C3—C4—N2	180.0
O1—C1—C2—Cl1	0.0		

### Hydrogen-bond geometry (Å, °)

**Table:**

*D*—H···*A*	*D*—H	H···*A*	*D*···*A*	*D*—H···*A*
C4—H4A···O1^i^	0.98 (2)	2.33 (2)	3.2988 (18)	171 (2)

Symmetry codes: (i) −*x*+1, −*y*+1/2, *z*+1/2.
